# Diagnosis of Alzheimer’s disease in asymptomatic individuals: a historical perspective

**DOI:** 10.1590/1980-5764-DN-2026-0487

**Published:** 2026-05-29

**Authors:** Ricardo Nitrini

**Affiliations:** 1 Universidade de São Paulo, Faculdade de Medicina, São Paulo SP, Brazil.

**Keywords:** Alzheimer Disease, Biomarkers, Diagnosis, Neurosyphilis, History, Dementia, Doença de Alzheimer, Biomarcadores, Diagnóstico, Neurossífilis, História, Demência

## Abstract

The diagnosis of Alzheimer’s disease (AD) based on biomarkers of the pathological process represents a significant change from previous criteria, which required the presence of dementia for AD diagnosis. These new criteria create difficulties in disclosing the diagnosis of AD to asymptomatic individuals. A similar example exists in the history of cognitive and behavioral disorders, where the risk of developing dementia paralytica (DP), a form of neurosyphilis (NS), could be detected even in the asymptomatic phase. Treatment in this phase of DP or in its early symptomatic phase was successful due to the discovery of new therapeutic modalities and new evolutionary biomarkers, which are not yet widely available in AD. This type of biomarker was very important in NS and will certainly be in AD, allowing for faster and less expensive clinical trials.

## RESUMO

O diagnóstico da doença de Alzheimer (DA) baseado em biomarcadores do processo patológico foi uma modificação muito grande em relação aos critérios anteriores, que exigiam a presença de demência para o diagnóstico da DA. Esses novos critérios criam dificuldades para revelar o diagnóstico de DA em indivíduos assintomáticos. Houve exemplo similar na história das doenças cognitivas e do comportamento, quando o risco de vir a ter paralisia geral progressiva (PGP), uma forma de neurossífilis (NS), tornou-se passível de detecção mesmo na fase assintomática. O tratamento nesta fase da PGP ou no início de sua fase sintomática foi bem-sucedido pela descoberta de novas modalidades terapêuticas e de novos biomarcadores evolutivos, que ainda não são amplamente disponíveis na DA. Esse tipo de biomarcador foi muito importante na NS e certamente será na DA, quando permitirá ensaios clínicos mais rápidos e de menor custo.

## INTRODUCTION

Diagnosing Alzheimer’s disease (AD), a relatively complex task, has become simpler with the change in the conception of AD and the development of plasma biomarkers. According to the recent AD criteria, the presence of dementia, or even the observation of any cognitive decline, is not necessary for diagnosing AD, because positive AD biomarkers demonstrate that the pathological process of AD has already begun^1^.

Not everyone accepted this new conception because Alzheimer’s disease (AD) had always been defined as clinicopathological entity where the presence of dementia was a *sine qua non* condition^2,3,4^. AD was and still is almost synonymous with “dementia due to AD” for most people^5^. According to the International Working Group, cognitively unimpaired individuals with positive biomarkers of Alzheimer’s pathology should be divided into two groups: asymptomatic at risk and presymptomatic (individuals at very high risk) depending on the biological stage of the disease^4^. To disclose the diagnosis of AD to an asymptomatic individual without a prior explanation of this new meaning of AD would be outrageous^5^.

Notwithstanding, in AD, as in any other degenerative disease, pathological changes begin before clinical manifestations. Early diagnosis of AD (or early diagnosis of at risk for AD dementia) has been possible for some years through biomarker analysis in cerebrospinal fluid (CSF)^6^. However, in clinical practice, spinal tapping was only performed for diagnosis in patients with cognitive decline of unclear etiology. In the asymptomatic phase, it was an exceptional procedure, reserved for research, for example, in patients with mutations that cause AD with dominant inheritance^7^.

With the approval of plasma biomarkers^8^, the possibility of early diagnosis of the pathological process of AD has become available and should become increasingly accessible in clinical practice.

In the last five years, treatments for AD in the mild cognitive impairment and mild dementia stages have been approved using monoclonal antibodies anti-beta-amyloid protein (mAb)^9,10,11^. The results obtained were statistically significant, but clinically very modest^12,13,14^. And results obtained with the treatment of asymptomatic AD have not been published yet.

Although this situation is new in AD, it is not new in medicine, and for this reason it may be worthwhile to evaluate similar conditions that have occurred in the past.

## EARLY DIAGNOSIS

Perhaps the medical field where early diagnosis is most important is oncology. Several types of cancer can be early diagnosed using various types of biomarkers and treated with complete success. Early diagnosis is also fundamental in infectious and immune-mediated diseases.

There is no doubt that diagnosis should precede treatment of any disease and that the earlier the diagnosis, the better the therapeutic outcome, particularly for brain pathological processes.

A very common type of dementia in the past went through a similar phase to what we are experiencing with AD: simpler diagnosis and lack of effective treatment.

## PARETIC NEUROSYPHILIS OR DEMENTIA PARALYTICA

Dementia paralytica (DP), also called general paralysis of the insane (GPI), a late form of neurosyphilis (NS), was probably the most common type of dementia in the last two centuries up to the first half of the 20^th^ century^15^.

In 1906, Wassermann reaction (WR) in the blood was described, and it started to be used to confirm or reject the diagnosis of syphilis in its early or late phases^16^.

A positive blood WR was associated with stigma of immoral behavior and intense fear of late forms of syphilis, particularly of the mental deterioration caused by DP^16^. (The low specificity of blood WR was not known in the initial years of its use, which certainly caused false alarms and serious problems to many people)^16^.

A few years before that, inflammatory changes in CSF were described in neurosyphilis, which were mainly characterized by pleocytosis (number of leukocytes >5 mm^3^) and high protein concentration. These inflammatory changes, especially when added to a positive WR in CSF, were the main CSF findings in DP and other forms of NS, which supported their diagnoses. Subsequently, it was described that these CSF inflammatory changes could be present in the latent phase of syphilis, a condition that was recognized as asymptomatic NS, which had a much stronger risk of progressing to symptomatic NS than a sole positive blood WR. Asymptomatic NS was present for many years before clinical manifestations, opening a window for preventive treatment^17,18^.

However, in the beginning of the 20^th^ century, the proposed treatments for syphilis had low efficacy for asymptomatic or symptomatic NS, and were even less efficacious for DP^18^.

That historic moment was considerably similar to that which we are living with regard to AD, with the difference that mAbs had shown statistically significant effects, although with very modest clinical effects^12,13,14^.

## WASSERMANN REACTION, A BIOMARKER OF EVOLUTION OF SYPHILIS

Although WR test was an indirect biomarker of syphilis, with both relatively low sensitivity and specificity, it had the great advantage of being a marker of the evolution of syphilis, i.e.: an evolutionary biomarker^16^. Titles of WR decreased in blood when early syphilis was treated with arsenicals (arsphenamines), which were introduced by Paul Ehrlich and Sahachiro Hata in 1910^16^. However, arsphenamines were not effective (or much less effective) in DP and other forms of NS^17^.

## MALARIOTHERAPY FOR DEMENTIA PARALYTICA

In 1917, Wagner von Jauregg initiated the treatment of DP with inoculation of *Plasmodium vivax* to cause several episodes of high fever, supposing that this would be curative for this disease. That treatment was successful, the adjective “irreversible” was excluded from the definition of dementia, and Wagner von Jauregg was distinguished with the Nobel prize in 1927^16^.

One of the most important findings of malariotherapy was that it was possible to know whether the treatment had been successful without waiting for months or years of neuropsychological and behavioral evaluations. When the treatment was effective, pleocytosis in CSF rapidly decreased to normal values (£5/mm^3^) after treatment. This evolutionary (or dynamic) biomarker was even more important than higher concentration of protein or titles of WR or in CSF, which take more time to decrease and may continue high or positive, respectively, for years after successful treatment^17,18^. However, malariotherapy had side effects, with mortality rates ranging from 1 to 20%^17^.

## PENICILLIN TREATMENT FOR DEMENTIA PARALYTICA

Treatment of DP with penicillin was initiated about three or four years after its discovery by Alexander Fleming in 1928^16^. The strong effect of penicillin in DP was easily demonstrated by the rapid reduction of pleocytosis in the CSF. Together with the effectiveness and low side effects, there was a rapid substitution of malariotherapy by penicillin in the treatment of DP. Alexander Fleming received the Nobel prize in 1945^16^. In DP, it was necessary to treat the symptoms as soon as they appeared, as this could halt the progression or even cause clinical resolution^19,20^. If treatment was carried out later, the progression of the disease was halted, but sequelae remained^21^.

Treatment of asymptomatic NS with penicillin, with careful follow-up to be sure that pleocytosis did not recur, cures the disease impeding the evolution to DP or to other forms of late NS^20^.

In conclusion, if the lights of the past can illuminate the present and the future to liberate us for creating new alternatives or pathways, there are many lessons we can learn from this brief history of DP.

As a first point, the possibility of diagnosing AD in the asymptomatic phase will be fundamental if treatment is able to prevent AD dementia. It is possible that treatment in the early symptomatic stages will also halt the progression of the disease.

We do not yet have this treatment, and it is not expected that a “silver bullet” will be discovered^22^, but rather that we will have multiple drugs with multiple targets^23^.

Another aspect relates to the sensitivity and specificity of the diagnosis. As mentioned, a positive result of blood WR created a stigma and a real fear of developing DP, but there were many false-positive results, and the majority of individuals, even those who were truly positive, did not develop DP.

In parallel, a positive plasma AD biomarker can cause stigma and fear of developing AD dementia. However, a positive plasma AD biomarker can have different prognoses, which we do not yet know precisely. For example, a positive plasma AD biomarker in an asymptomatic individual or one with subjective cognitive decline probably has different prognoses, for example, if the individual is aged 50 or 80 years. And many individuals with positive plasma AD biomarkers will not progress to AD dementia during their lifetimes^3,4,24^. These facts should be presented before requesting plasma AD biomarkers, followed by careful discussion with patient after positive results. Plasma biomarkers should be understood, by doctors and patients, as having a probabilistic value regarding evolution to AD dementia^24^.

In asymptomatic NS and DP, treatment has been based on an important evolutionary biomarker: pleocytosis in the CSF. That type of evolutionary biomarker is not yet available for the pathology of AD. Therefore, we need to rely on neuropsychological tests and functional assessments that take a long time to change. Tau positron emission tomography (PET-Tau) is currently the best biomarker for the pathological stage of AD, but it is not widely available and was not shown to be a reliable evolutionary biomarker in the study that led to the approval of the treatment with donanemab^11^.

It is already known that p-tau217 progressively increases in CSF or plasma with increasing disease severity^25^. It is possible that this biomarker or others, like biomarkers of synaptic degeneration^23^, could be evolutionary markers of AD. Based on history and confidence in scientific development, we will soon have reliable and simpler evolutionary biomarkers for AD, which will allow shorter and less expensive clinical trials. That will lead to new effective treatments.

## Data Availability

The datasets generated and/or analyzed during the current study are available from the corresponding author upon reasonable request.
